# Investigating cell-specific effects of FMRP deficiency on spiny projection neurons in a mouse model of Fragile X syndrome

**DOI:** 10.3389/fncel.2023.1146647

**Published:** 2023-05-30

**Authors:** Gabriele Giua, Olivier Lassalle, Leila Makrini-Maleville, Emmanuel Valjent, Pascale Chavis, Olivier J. J. Manzoni

**Affiliations:** ^1^INMED, INSERM U1249, Marseille, France; ^2^Aix-Marseille University, Marseille, France; ^3^Cannalab “Cannabinoids Neuroscience Research International Associated Laboratory”, INSERM-Aix-Marseille University/Indiana University, Marseille, France; ^4^IGF, University of Montpellier, INSERM, CNRS, Montpellier, France

**Keywords:** Fragile X, accumbens, D1R, D2R, spiny projection neurons

## Abstract

**Introduction:**

Fragile X syndrome (FXS), resulting from a mutation in the *Fmr1* gene, is the most common monogenic cause of autism and inherited intellectual disability. *Fmr1* encodes the Fragile X Messenger Ribonucleoprotein (FMRP), and its absence leads to cognitive, emotional, and social deficits compatible with the nucleus accumbens (NAc) dysfunction. This structure is pivotal in social behavior control, consisting mainly of spiny projection neurons (SPNs), distinguished by dopamine D1 or D2 receptor expression, connectivity, and associated behavioral functions. This study aims to examine how FMRP absence differentially affects SPN cellular properties, which is crucial for categorizing FXS cellular endophenotypes.

**Methods:**

We utilized a novel *Fmr1−/y*::*Drd1a-tdTomato* mouse model, which allows *in-situ* identification of SPN subtypes in FXS mice. Using RNA-sequencing, RNAScope and *ex-vivo* patch-clamp in adult male mice NAc, we comprehensively compared the intrinsic passive and active properties of SPN subtypes.

**Results:**

*Fmr1* transcripts and their gene product, FMRP, were found in both SPNs subtypes, indicating potential cell-specific functions for *Fmr1*. The study found that the distinguishing membrane properties and action potential kinetics typically separating D1- from D2-SPNs in wild-type mice were either reversed or abolished in *Fmr1−/y*::*Drd1a-tdTomato* mice. Interestingly, multivariate analysis highlighted the compound effects of *Fmr1* ablation by disclosing how the phenotypic traits distinguishing each cell type in wild-type mice were altered in FXS.

**Discussion:**

Our results suggest that the absence of FMRP disrupts the standard dichotomy characterizing NAc D1- and D2-SPNs, resulting in a homogenous phenotype. This shift in cellular properties could potentially underpin select aspects of the pathology observed in FXS. Therefore, understanding the nuanced effects of FMRP absence on SPN subtypes can offer valuable insights into the pathophysiology of FXS, opening avenues for potential therapeutic strategies.

## Introduction

Fragile X Syndrome (FXS) is caused by meiotic instability in the 5′ untranslated region of the X chromosome-linked Fragile X messenger ribonucleoprotein 1 gene (*Fmr1*), impeding the transcription of the Fragile X Messenger Ribonucleoprotein (FMRP) (Suardi and Haddad, 2020). FMRP is an RNA-binding protein, widely expressed in the central nervous system, which regulates the translation of thousands of mRNA targets and thus its loss in FXS largely modifies protein synthesis (Darnell et al., 2011; Ascano et al., 2012; Tabet et al., 2016). Among the pleiotropic outcomes of FMRP absence, altered neuronal and circuit excitability is thought to be a major component of FXS phenotypes (Contractor et al., 2015). The variety of neuropsychiatric symptoms in FXS, such as learning disabilities, hyperactivity, repetitive behaviors, impulsivity, and symptoms affecting social behaviors associated with autism spectrum disorder, suggests dysfunction in multiple brain areas. Cortical (Selby et al., 2007; Gibson et al., 2008; Hays et al., 2011; Testa-Silva et al., 2012; Haberl et al., 2015; Martin et al., 2016, 2017; Kramvis et al., 2020) and hippocampal (Bostrom et al., 2016) dysfunctions have been extensively studied. Although the symptomology of FXS includes social deficits (Cregenzán-Royo et al., 2022) compatible with altered neuronal functions in the mesolimbic network and the basal ganglia (Supekar et al., 2018; Pfaff and Barbas, 2019), studies addressing how FXS modifies neuronal excitability in these systems are scarce. The central part of the mesolimbic pathway, the nucleus accumbens (NAc), processes emotional, motivational, reward and aversion signals and plays a role in behaviors essential to the survival of the animal (Bromberg-Martin et al., 2010; Floresco, 2015). The lack of FMRP modifies synaptic long-term potentiation (Neuhofer et al., 2015), long-term depression (Jung et al., 2012; Neuhofer et al., 2018) in the NAc as well as the dendritic morphology of the principal cell-type of the NAc, spiny projection neurons (SPNs) (Neuhofer et al., 2015). NAc SPNs are GABAergic projection neurons which express either D1 or D2 receptors (D1R or D2R) and play specific roles in NAc-mediated behaviors and disorders (Lobo and Nestler, 2011; Francis et al., 2015). Several common symptoms of autism spectrum disorders observed in FXS indicate an altered balance between approach and avoidance responses. Specifically, direct pathway D1-SPNs mediate approach behavioral responses while D2-SPNs mediate avoidance behavioral responses via the so-called indirect pathway (Pfaff and Barbas, 2019). Here we explored the cell-type specific intrinsic properties of SPNs in the NAc Core of a novel *Fmr1*+*/y* or *−/y*: *Drd1a-tdTomato* mouse model allowing *in situ* identification of SPN subtypes in wild type (WT) and FXS littermate mice. Overall, the results show the cell-specific endophenotypes of FMRP’s ablation in the NAc: the normal dichotomy that characterizes D1- and D2-SPNs is thrown out of balance, leading to a uniform phenotype that could underlie selected aspects of the pathology.

## Materials and methods

### Animals

**FIGURE 1:**
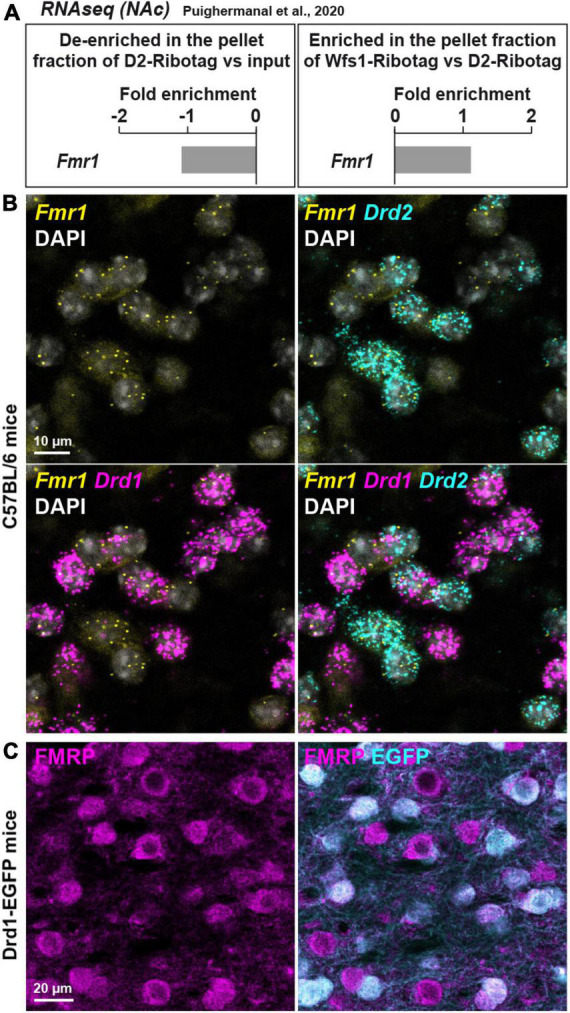
*Fmr1* mRNA is detected in D1- and D2-SPNs in the NAc Core of WT mice. **(A)** Fold-change of *Fmr1* transcripts found by RNAseq in NAc extract of D2-Ribotag and Wfs1-Ribotag mice (Puighermanal et al., 2020). *Fmr1* transcripts were slightly “de-enriched” in the NAc pellet fraction of *D2-RiboTag* mice compared with the input fraction (containing the mRNAs from all cellular types) (left panel) but slightly enriched in the NAc pellet fraction of *Wfs1-RiboTag* mice (right panel). **(B)** Single-molecular fluorescent *in situ* hybridization for *Drd1* (magenta), *Drd2* (cyan), and *Fmr1* (yellow) mRNAs in the NAc Core. Slides were counterstained with DAPI (white). Scale bar, 10 μm. *Fmr1* mRNA expression is detected in both *Drd1*-positive and *Drd2*-positive neurons. **(C)** Double immunofluorescence for FMRP (magenta) and EGFP (cyan) in the NAc Core of *Drd1*-EGFP mice. Scale bars, 20 μm.

**FIGURE 2:**
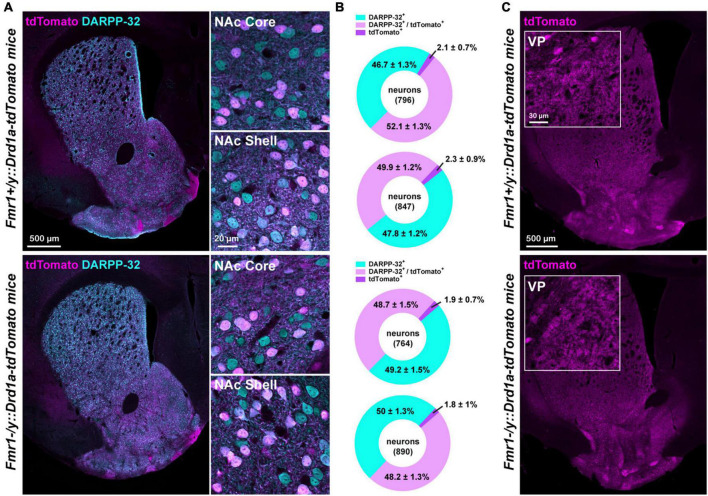
Proportion of D1- and D2-SPNs in FXS mice. **(A)** Double immunofluorescence for tdTomato (magenta) and DARPP-32 (cyan) in the striatum (left) of *Fmr1*+*/y: Drd1a-tdTomato* (upper panel) and *Fmr1–/y: Drd1a-tdTomato* (lower panel) mice. High magnification in the NAc Core and Shell (right). **(B)** Charts show the proportion of D1- and D2-SPNs in the NAc Core and Shell of *Fmr1*+*/y: Drd1a-tdTomato* (upper) and *Fmr1–/y*: *Drd1a-tdTomato* mice (lower). Quantifications were obtained from four images per region of five mice of each genotype. **(C)** Single immunofluorescence for tdTomato reveals axonal projections from D1-SPNs that terminate in the ventral pallidum (VP) in *Fmr1*+*/y: Drd1a-tdTomato* (upper panel) and *Fmr1–/y: Drd1a-tdTomato* (lower panel) mice. Insets: high magnification of the terminals in the VP.

Animals were treated in compliance with the European Communities Council Directive (86/609/EEC) and the United States National Institutes of Health Guide for the care and use of laboratory animals. The French Ethical committee authorized this project (APAFIS#3279-2015121715284829 v5). Three different cohorts of mice were used in this study. (1) Mice implied in electrophysiological experiments were obtained breeding *Drd1a-tdTomato* × *Fmr1* KO2 mice from the Jackson Laboratory (Bar Harbor, ME, USA) and FRAXA foundation, respectively. Both strains had a C57Bl/6J background. Mice were acclimated to the animal facility for 1 week and then housed in male *Drd1a-tdTomato* and female *Fmr1*± pairs for breeding. Pups were weaned and ear punched for identification and genotyping at P21. *Ex vivo* electrophysiological recordings were performed on first-generation male mice between P70 and P100. *Fmr1*+*/y*: *Drd1a-tdTomato* mice composed the control group WT and *Fmr1−/y*: *Drd1a-tdTomato* the experimental group [knockout (KO)]. This genetic line was also employed for the immunofluorescence experiments described in Figure 2. (2) For immunofluorescence experiments described in Figure 1 we used a cohort of 8–12-week-old male mice *Drd1a*-*EGFP* (enhanced green fluorescent protein) (*n* = 3, C57BL/6 background, founder *S118*), generated by GENSAT (Gene Expression Nervous System Atlas, Rockefeller University, New York, NY, USA). (3) Male C57BL/6J mice 8 weeks old from Jackson Laboratory were used for *in situ* hybridization (ISH) assay (*n* = 2). All mice used in this study were housed in groups of 4–5 mice at constant room temperature (20 ± 1°C) and humidity (60%) and exposed to a light cycle of 12 h light/dark with *ad libitum* access to food and water.

### Immunofluorescence assay

Mice were rapidly anesthetized with Euthasol (360 mg/kg, i.p., TVM Lab, France) and transcardially perfused with 4% (weight/vol) paraformaldehyde in 0.1 M sodium phosphate buffer (PBS) (pH 7.5) (Gangarossa et al., 2013). Brains were post-fixed overnight at 4°C in the same solution. Thirty-micrometer thick sections were cut with a vibratome (Leica, France) and stored at −20°C in a solution containing 30% (vol/vol) ethylene glycol, 30% (vol/vol) glycerol, and 0.1 M sodium phosphate buffer, until they were processed for immunofluorescence. NAc sections were identified using a mouse brain atlas and sections comprised between 1.54 and 1.42 mm from bregma were included in the analysis. Free-floating sections were rinsed three times 10 min in PBS followed by an incubation of 15 min in 0.2% (vol/vol) Triton X-100 in PBS. Sections were rinsed again in PBS and blocked for 1 h in a solution of 3% BSA in PBS, before being incubated 72 h at 4°C with the following primary antibodies: chicken anti-GFP (1:1,000, Life Technologies, #A10262), rabbit anti-FMRP (1:500, Millipore, #ab60-46), mouse anti-RFP (1:1,000, MBL, #M155-3), and rabbit anti-DARPP32 (1:1,000, Cell Signaling Technology, #2306). Sections were rinsed three times for 10 min in PBS and incubated for 45 min with goat Cy3-coupled anti-rabbit (1:500, Thermo Fisher Scientific, Cat. #10520), goat Alexa Fluor 488-coupled anti-chicken (1:500, Thermo Fisher Scientific, Cat. #A-11039), goat Alexa Fluor 594-coupled anti-mouse (1:500, Thermo Fisher Scientific, Cat. #A-11005), and goat Alexa Fluor 488-coupled anti-rabbit (1:500, Thermo Fisher Scientific, Cat. #A-11034). Finally, sections were rinsed for 10 min twice in PBS before mounting in DPX (Sigma-Aldrich).

### RNAscope ISH assay

Staining for *Drd1* (dopamine receptor D1 gene), *Drd2* (dopamine receptor D2 gene), and *Fmr1* mRNAs was performed using single molecule fluorescent *in situ* hybridization (smFISH). Brains from 2 C57BL/6J (8 weeks old) male mice were rapidly extracted and snap-frozen on dry ice and stored at −80°C until use. Ventral striatum coronal sections (14 μm) were collected directly onto Superfrost Plus slides (Fisherbrand). RNAscope Fluorescent Multiplex labeling kit (ACDBio, Cat No. 320850) was used to perform the smFISH assay according to manufacturer’s recommendations. Probes used for staining are mm-*Drd1*-C1 (ACDBio, Cat No. 461901), mm-*Drd2*-C3 (ACDBio, Cat No. 406501-C3), and mm-*Fmr1*-C2 (ACDBio, Cat No. 496391-C2). After incubation with fluorescent-labeled probes, slides were counterstained with DAPI and mounted with ProLong Diamond Antifade mounting medium (Thermo Fisher Scientific, P36961).

### Images acquisition

Confocal microscopy and image analysis were carried out at the Montpellier RIO Imaging Facility. Images covering the entire striatum and double-labeled images from each region of interest were acquired using sequential laser scanning confocal microscopy (Leica SP8). Photomicrographs were obtained with the following band-pass and long-pass filter setting: Alexa Fluor 488 (band pass filter: 505–530), Alexa Fluor 594/Cy3 (band pass filter: 560–615), and Cy5 (long-pass filter: 650). All parameters were held constant for all sections from the same experiment. Two slices per mouse were used for quantification. Adjacent serial sections were never counted to avoid any potential double counting of hemisected neurons.

### Slice preparation for *ex vivo* electrophysiological recordings

Adult male mice (P70–P100) were deeply anesthetized with isoflurane and decapitated according to institutional regulations, as previously described (Deroche et al., 2020). The brain was sliced (300 μm) on the coronal plane with a vibratome (Integraslice, Campden Instruments) in a sucrose-based solution at 4°C (NaCl 87 mM, sucrose 75 mM, glucose 25 mM, KCl 2.5 mM, MgCl_2_ 4 mM, CaCl_2_ 0.5 mM, NaHCO_3_ 23 mM, and NaH2PO_4_ 1.25 mM). Immediately after cutting, slices containing the NAc Core were stored for 1 h at 32°C in a low calcium artificial cerebrospinal fluid (ACSF; NaCl 130 mM, glucose 11 mM, KCl 2.5 mM, MgCl_2_ 2.4 mM, CaCl_2_ 1.2 mM, NaHCO_3_ 23 mM, and NaH_2_PO_4_ 1.2 mM), equilibrated with 95% O_2_/5% CO_2_. After 1 h of recovery, slices were kept at room temperature until the time of recording.

### Electrophysiology

Whole-cell patch-clamp recordings were collected from SPNs of NAc Core. SPNs were visualized using an upright microscope with infrared illumination and then distinguished in D1 or D2 expressing SPNs based on the visualization of *Drd1*-tdTomato using an upright microscope with infrared and fluorescent illumination. During the recording, coronal slices containing the NAc were placed in the recording chamber and superfused at 2 ml/min with normal calcium ACSF (NaCl 130 mM, glucose 11 mM, KCl 2.5 mM, MgCl_2_ 1.2 mM, CaCl_2_ 2.4 mM, NaHCO_3_ 23 mM, and NaH_2_PO_4_ 1.2 mM), equilibrated with 95% O_2_/5% CO_2_. The intracellular solution was based on K^+^ gluconate (K^+^ gluconate 145 mM, NaCl 3 mM, MgCl_2_ 1 mM, EGTA 1 mM, CaCl_2_ 0.3 mM, Na_2_ATP 2 mM, NaGTP 0.5 mM, cAMP 0.2 mM, buffered with HEPES 10 mM). Its pH was adjusted to 7.2 and osmolarity to 290–300 mOsm. Electrode resistance was 3–5 MOhm. All experiments were done at 25 ± 1°C. The superfusion medium contained gabazine 10 μM (SR 95531 hydrobromide; Tocris) to block GABA Type A (GABA-A) receptors.

Data was recorded in current clamp with an Axopatch-200B amplifier, low pass filtered at 2 kHz, digitized (10 kHz, DigiData 1440A, Axon Instruments), collected and analyzed using Clampex 10.7 (Molecular Device).

**FIGURE 3:**
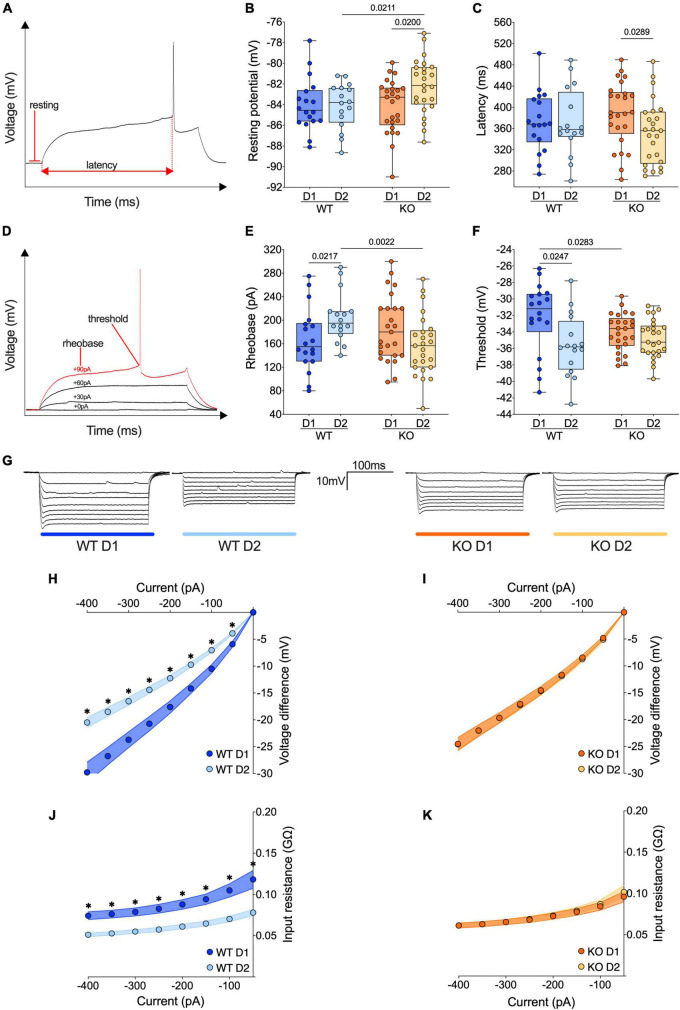
The passive properties of D1- and D2-SPNs equalize in FXS mice. **(A)** Example of individual AP evoked by depolarizing current injection, indicating resting potential, and latency metrics. **(B)** The resting membrane potential of D2-SPNs is significantly depolarized in *Fmr1* KO mice compared to that of D2-SPNs of WT mice and D1-SPNs recorded in *Fmr1* KOs. **(C)** In *Fmr1* KO mice D2-SPNs showed shorter firing latency than D1-SPNs. **(D)** Example of individual AP evoked by increasing current steps, indicating rheobase and action potential threshold. **(E)** In WT mice the rheobase is higher in D2-SPNs than in D1-SPNs but in *Fmr1* KO mice the rheobases are similar. **(F)** In WT mice, D1-SPNs have a higher spiking threshold than D2-SPNs. In contrast, this difference is absent in *Fmr1* KO. **(B,C,E,F)** Single dot represents an individual mouse. Data are shown as min. to max. box plot with median and 25–75 percentile. Mann–Whitney *U* tests. *p*-Values <0.05 are displayed in graphs. **(G)** Examples of membrane voltage response to hyperpolarizing current injections in our experimental groups. **(H)** Current injection steps of 50 pA from –400 to –50 pA revealed differences in the I–V relationship in D1- and D2-SPNs in the accumbens of WT mice (i.e., in response to hyperpolarizing current injections, in WT mice D1-SPNs showed a greater response compared to D2-SPNs), **(I)** but not *Fmr1* KO mice. **(J)** The membrane input resistance was higher in D1-SPNs than in D2-SPNs in WT, **(K)** but not *Fmr1* KO mice. **(H–K)** Single dot represents group mean value at that current step. Data are shown as mean ± SEM in XY plot. Multiple Mann–Whitney *U* test, *p-values <0.05. **(B,C,E,F,H–K)** WT D1-SPNs *N* = 18 in dark blue, WT D2-SPNs *N* = 16 in light blue, KO D1-SPNs *N* = 25 in dark orange, and KO D2-SPNs *N* = 25 in light orange.

**FIGURE 4:**
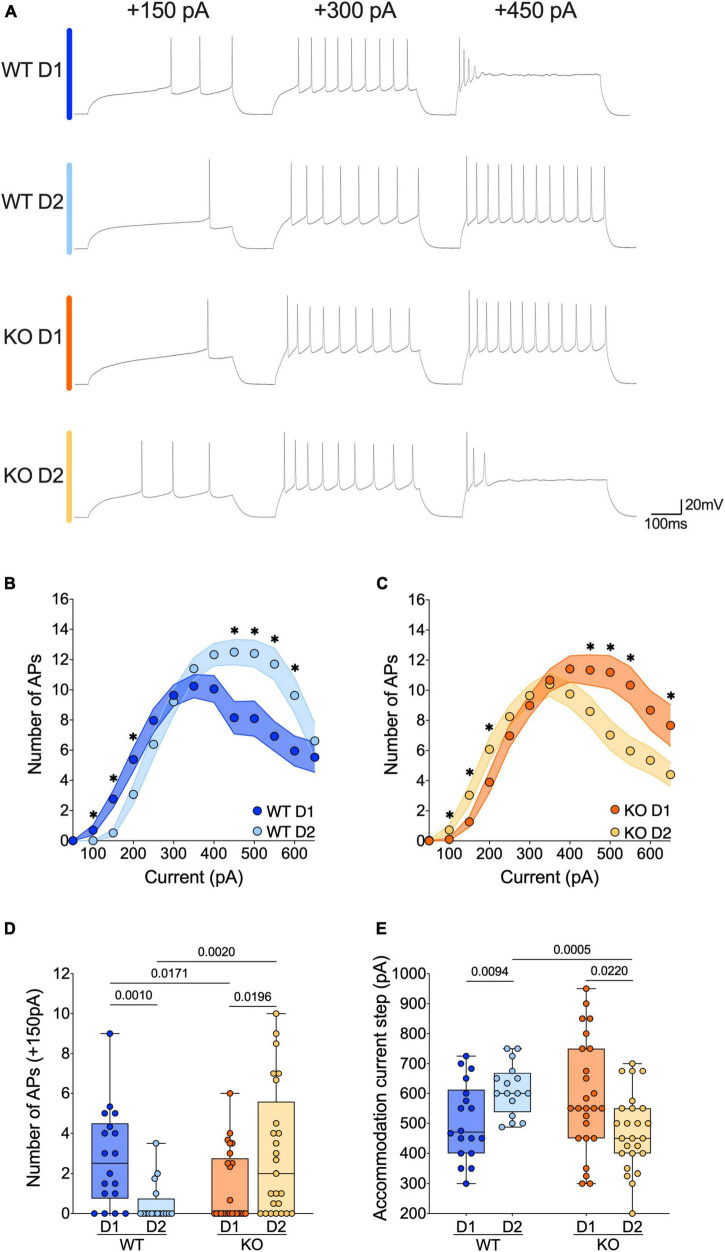
The active properties of SPNs’ subtypes diverge in opposite ways in WT and FXS mice. **(A)** Sample spike trains in response to depolarizing currents from D1- and D2-SPNs in each genotype. **(B,C)** Step by step analysis of the number of evoked action potentials in response to increasing depolarizing current shows that the hierarchy of excitability between D1- and D2-SPNs reverses in *Fmr1* KO mice. Single dot represents group mean value for each current step. Data are shown as mean ± SEM in XY plot. Multiple Mann–Whitney *U* test, *p-values < 0.05. **(D)** At physiological depolarizing current steps, D1-SPNs are more excitable than D2-SPNs in WT mice, whereas in *Fmr1* KO mice, D1-SPNs are less excitable than D2-SPNs. **(E)** In WT mice the D1-SPNs accommodate their firing at lower current steps compared with D2-SPNs, while it is the exact opposite in *Fmr1* KO mice. **(D,E)** Single dot represents an individual mouse. Data are shown as min. to max. box plot with median and 25–75 percentile. Mann–Whitney *U* tests. *p*-Values < 0.05 are displayed in graphs. **(B–E)** WT D1-SPNs *N* = 18 in dark blue, WT D2-SPNs *N* = 16 in light blue, KO D1-SPNs *N* = 25 in dark orange, and KO D2-SPNs *N* = 25 in light orange.

**FIGURE 5:**
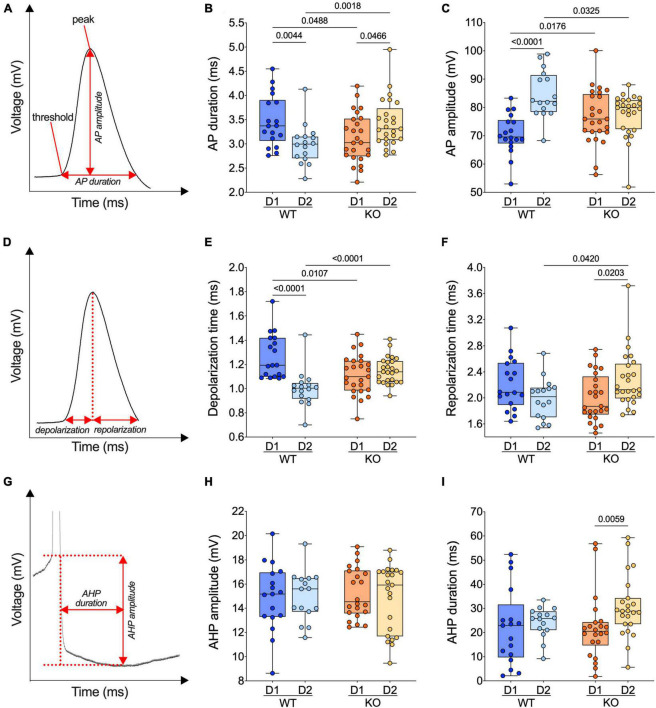
The normal dichotomy of action potential properties of SPNs’ subtypes reverts in FXS mice. **(A)** Example of individual AP evoked by depolarizing current injection, showing its threshold, peak, amplitude, and duration. **(B)** In WT mice, APs are shorter in D2- than D1-SPNs whereas in *Fmr1* KO mice, APs are longer in D2- than D1-SPNs. **(C)** In WT mice, APs are larger (i.e., the voltage difference between threshold and peak) in D2- than D1-SPNs but similar in *Fmr1* KO mice. **(D)** Example of individual AP evoked by depolarizing current injection, indicating depolarization and repolarization metrics. **(E)** AP depolarization time is shorter in D2- than in D1-SPNs in WT and similar in *Fmr1* KO mice. **(F)** The AP repolarization time of D2-SPNs is shorter in WT than *Fmr1* KO mice. **(G)** Example of individual AP evoked by depolarizing current injection, indicating AHP amplitude and AHP duration metrics. **(H,I)** AHP amplitude is comparable in all groups and its duration selectively increased in D2-SPNs from *Fmr1* KO mice. **(B,C,E,F,H,I)** Single dot represents an individual mouse. Data are shown as min. to max. box plot with median and 25–75 percentile. Mann–Whitney *U* tests. *p*-Values <0.05 are displayed in graphs. **(B,C,E,F)** WT D1-SPNs *N* = 18 in dark blue, WT D2-SPNs *N* = 16 in light blue, KO D1-SPNs *N* = 25 in dark orange, and KO D2-SPNs *N* = 25 in light orange. **(H,I)** WT D1-SPNs *N* = 17 in dark blue, WT D2-SPNs *N* = 15 in light blue, KO D1-SPNs *N* = 22 in dark orange, and KO D2-SPNs *N* = 23 in light orange.

During a current clamp protocol, resting membrane potential, membrane voltage response, input resistance, the number of action potentials (APs), and the accommodation current step were determined by applying current steps ranging from −400 to +900 pA in increments of +50 pA, each lasting 500 ms. Using a current clamp protocol ranging from 0 pA to the rheobase in +10 pA increments, each lasting 500 ms, rheobase, latency, threshold, action potential (AP) properties, afterhyperpolarization (AHP) amplitude, and duration were analyzed for the first AP at the rheobase step. When recordings were obtained from the same mouse, data from multiple neurons (1–3) of the same cell type were averaged. Resting membrane potential was assessed at the beginning of the whole-cell recording during the current clamp protocol (Figure 3A). The membrane voltage response was evaluated based on the steady-state voltage during hyperpolarizing current injections (Figure 3G). Input resistance was computed as the change in membrane voltage (ΔmV) divided by the injected current (pA). The number of APs was determined for each depolarizing current step lasting 500 ms. The accommodation current step was identified as the depolarizing current step at which the neuron begins to fail in evoking APs (e.g., in Figure 4A at +450 pA). Rheobase was defined as the minimum current necessary to elicit an AP (Figure 3D). Latency referred to the time delay in triggering the first AP during the rheobase current step (Figure 3A). The threshold was determined as the point where the depolarization slope dramatically changes, signifying AP initiation (Figures 3D, 5A). AP duration was calculated by measuring the time taken for the voltage to travel from the threshold to the equipotential point during the repolarization phase (Figure 5A), which was further divided into depolarization and repolarization times based on the AP peak (Figures 5A, D). AP amplitude was computed as the peak voltage minus the threshold voltage (Figure 5A). The afterhyperpolarization (AHP) amplitude was assessed as the voltage difference between the threshold and the minimum voltage recorded during AHP (Figure 5G). Finally, AHP duration was measured as the time from the end of AP repolarization (equipotential point of the threshold) to the AHP peak (Figure 5G).

### Statistical analysis

Data were tested for Normality (D’Agostino–Pearson and Shapiro–Wilk tests) and outliers (ROUT test) before statistical analysis. Non-parametric Mann–Whitney *U* and Spearman correlation tests were performed with Prism (GraphPad Software). Correlations were compared with “cocor.indep.groups” of the package cocor (Diedenhofen and Musch, 2015) in R 4.2.0 (R Core Team, 2020). Comparison results were shown only for those correlations found to be significant in at least one of the two compared groups. The index was built with input resistance, APs number at +150 pA current step and AP duration, which reflect passive membrane properties, neuronal firing properties and APs kinetics, respectively. The values were standardized for each variable by scaling (mean of 0 and SD of 1) and pooled for each group. *N* values represent individual animals. Statistical significance was set at *p* < 0.05.

## Results

We recorded a total of 156 SPNs from the NAc Core of 49 *Fmr1*+*/y* or *−/y: Drd1a-tdTomato* male mice (P70–P100). Given that in this specific mouse line, tdTomato-unlabeled SPNs display a very low contamination rate (ranging from approximately 1.9% in the dorsal striatum to about 7.3% in the NAc Core) with Drd1a-positive SPNs (Gagnon et al., 2017), it can be reasonably deduced that almost all unlabeled D1-negative SPNs are D2-positive SPNs. For ease of understanding, we will refer to tdTomato-unlabeled SPNs as D2-SPNs throughout this study, while recognizing that this classification is tentative. The dataset was divided into four groups: *tdTomato*-labeled *Drd1a*-positive SPNs from WT mice (WT D1), *tdTomato*-unlabeled SPNs from WT mice (WT D2), *tdTomato*-labeled *Drd1a*-positive SPNs from *Fmr1* KO mice (KO D1), and *tdTomato*-unlabeled SPNs from *Fmr1* KO mice (KO D2). The analysis was developed to highlight: (i) differences between SPN subtypes within each genotype; (ii) differences between the two genotypes for the same SPN subtype.

### *Fmr1* mRNA is expressed in both D1- and D2-SPNs in the NAc Core of WT mice

First, the expression of *Fmr1* transcripts was quantified in a dataset of RNAseq (Gene Expression Omnibus, GSE94145) generated on tagged ribosome-bound mRNAs and the input fractions of NAc extract (including both Core and Shell) of 10-week-old D2-Ribotag and Wfs1-Ribotag mice (Puighermanal et al., 2020). *Fmr1* transcripts were less abundant (adjusted *p*-value <0.05) in the NAc pellet fraction of D2-Ribotag mice compared with the input fraction (containing the mRNAs from all cellular types) suggesting that *Fmr1* gene products were less expressed in D2-SPNs (Figure 1A, left). These results were in line with the slight enrichment of *Fmr1* transcripts in the NAc pellet fraction of Wfs1-SPNs, a NAc Core SPNs population enriched in *Drd1* transcripts (Puighermanal et al., 2020; Figure 1A, right). The presence of *Fmr1* mRNA in NAc Core SPNs was confirmed by *in situ* hybridization. Indeed, *Fmr1* transcripts were found in striatal cells expressing the gene encoding for the dopamine D1 and D2 receptors (*Drd1* and *Drd2*), identifying D1- and D2-SPNs, respectively (Figure 1B). The distribution of FMRP in both SPNs population was finally validated at the protein level. Double immunofluorescence analysis of the NAc Core of Drd1-EGFP mice revealed that FMRP was detected in both GFP-positive (D1-SPNs) and GFP-negative (D2-SPNs) (Bertran-Gonzalez et al., 2008; Figure 1C). Together, these results indicate that both NAc Core D1- and D2-SPNs express FMRP.

### Distribution of D1- and D2-SPNs in the NAc Core of WTs and FXS mice

To determine whether the proportion of D1- and D2-SPNs in the NAc is preserved between the WT and FXS mice, the percentage of tdTomato-positive cells co-expressing the dopamine- and cAMP-regulated phosphoprotein Mr∼32,000 (DARPP-32), a marker of all SPNs, was estimated in the NAc Core and Shell (Figures 2A, B). The proportion of D1-SPNs (tdTomato^+^/DARPP-32^+^) and D2-SPNs (tdTomato^–^/DARPP-32^+^) in the NAc was found to be similar between the WT and FXS mice (Figure 2B). Similar results were found for the fraction of tdTomato^+^/DARPP-32^–^ (∼2% of the cells quantified), which most likely correspond to striatal GABAergic interneurons expressing either tyrosine hydroxylase (Ibáñez-Sandoval et al., 2015) of the neuropeptide Y (known as neurogliaform) (Tepper et al., 2018). Finally, the analysis of tdTomato-labeled terminals in the ventral pallidum confirmed that axonal projections in the major projection nucleus of NAc Core D1-SPNs were not altered in FXS mice (Figure 2C).

### Discrepancies in the passive properties of accumbal D1- and D2-SPNs in WTs vanishes in FXS mice

The passive properties of adult current-clamped and visually identified neighboring D1 and D2 SPNs were compared in the NAc Core of WT and *Fmr1* KO littermates (Figure 3 and Supplementary Figure 1). In WT mice, the resting potential of D1- and D2-SPNs is similar. In contrast, D2-SPNs were more depolarized than D1-SPNs in *Fmr1* KO mice (Figure 3B). No differences in the firing latency were observed in WTs while in *Fmr1* KO mice D2-SPNs fired more readily than D1-SPNs (Figure. 3C). In WT mice, D2-SPNs displayed a higher rheobase compared to D1-SPNs whereas in *Fmr1* KO this difference disappeared. Here, D2-SPNs of *Fmr1* KO mice showed a lower rheobase when compared to D2-SPNs of WTs, according to their depolarized state (Figure 3E). Moreover, D2-SPNs exhibited a more negative threshold than D1-SPNs in WTs and this dichotomy vanished in *Fmr1* KO mice. Differentiating cell subtypes showed that only D1-SPNs varied between genotypes, with a more negative threshold in *Fmr1* KO mice (Figure 3F). As shown in Figure 3G, the membrane voltage responses to hyperpolarizing current steps strongly differed between D1- and D2-SPNs in WTs (Figure 3H) but were similar in *Fmr1* KO mice (Figure 3I). This difference likely reflects an alteration in input resistance in the absence of FMRP (Figures 3J, K and Supplementary Figures 1D, E). Specifically, in *Fmr1* KO mice the input resistance of D1-SPNs was decreased (Supplementary Figure 1D) whereas that of D2-SPNs was increased (Supplementary Figure 1E) when compared with WT. Finally, the membrane response to a somatic injection of −400 pA also revealed a higher sag current in the D2-SPNs of *Fmr1* KO mice when compared with those of WTs (Supplementary Figure 1G). Taken together, these results show that the profound functional differences in passive properties that normally differentiate D1- from D2-SPNs are abolished in mice lacking FMRP.

### The active properties of accumbal D1- and D2-SPNs diverge in opposite ways in WT and FXS mice

The current changes in passive properties strongly suggest cell-subtype specific modifications of SPNs’ intrinsic excitability. The membrane reaction profiles of D1- and D2-SPNs in response to a series of increasing somatic current steps differ strongly between the two genotypes (Figures 4A–C). Within physiological range, D1- were more excitable than D2-SPNs in WT mice, whereas in *Fmr1* KO mice it was the opposite (Figures 4A–D). Conversely, at higher current steps, firing accommodation occurred at lower current steps in D1- than in D2-SPNs in WTs, contrary to KO (Figure 4E). The comparison of firing profile by cell types showed that the absence of FMRP impacted the excitability of both neuronal subtypes in opposite manners (Supplementary Figures 2A–E), in parallel with opposite changes in input resistance (Supplementary Figures 1D, E).

### Divergent action potential properties of D1- and D2-SPNs in WT and FXS mice

Given the cell-specific impact of the absence of FMRP on the intrinsic excitability of SPNs, action potential kinetics were examined in our different cell types and genotypes. Analysis of AP duration (Figure 5A) revealed that APs in D1-SPN were longer than that of D2-SPNs in WT, whereas an opposite dichotomy was found in *Fmr1* KO mice (Figure 5B). Specifically, ablation of *Fmr1* widened and shortened APs in D2-SPNs and D1-SPNs, respectively (Figure 5B). The loss of the normal dichotomy in AP duration was paralleled by opposite changes in AP amplitude of D1-SPNs and D2-SPNs in the absence of FMRP: compared to WT, APs got larger in D1- and smaller in D2-SPNs (Figure 5C). We reasoned that the observed differences in AP duration could result from phase specific changes in the depolarization and/or repolarization period (Figure 5D). In WTs, the AP depolarization phase was longer in D1- than in D2-SPNs; this physiological divergence disappeared in *Fmr1* KO mice (Figure 5E). AP repolarization times were similar across genotypes in D1-SPNs but not in D2-SPNs that repolarized slower in *Fmr1* KO mice (Figure 5F). Finally, comparing the AP undershoot phase, the so-called afterhyperpolarization (AHP) (Figure 5G), showed that while AHP amplitudes were similar in all groups (Figure 5H), AHPs were longer in D2-SPNs from *Fmr1* KO mice (Figure 5I).

### The normal dichotomy that characterizes D1- and D2-SPNs is thrown out of balance in absence of FMRP

**FIGURE 6:**
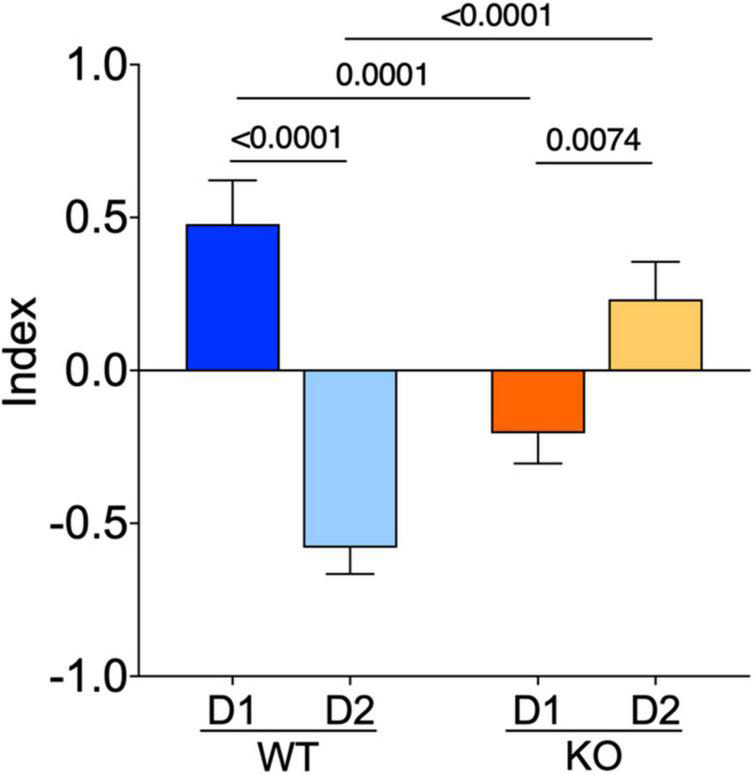
The normal dichotomy characterizing D1- and D2-SPNs is thrown out of balance in *Fmr1* KO. D1- and D2-SPNs exhibit a dichotomy of their electrophysiological profile which is reversed in KO mice. FMRP’s absence impacts SPNs subtypes in opposite manners. The index combines input resistance, APs number at +150 pA and APs duration parameters. Data were standardized per variable and plotted per group. Data are shown as mean with SEM column bar graph. Mann–Whitney *U* tests. *p*-Values <0.05 are displayed in graphs. WT D1-SPNs *N* = 18, WT D2-SPNs *N* = 16, KO D1-SPNs *N* = 25, and KO D2-SPNs *N* = 25 for each plotted variable.

To summarize our comparative electrophysiological profiling, an index based on passive properties, excitability, and APs kinetics of these neurons was computed. The index reflected the strong dichotomy between D1- and D2-SPNs present in WTs. In *Fmr1* KO mice the dichotomy changed polarity, likely because of opposite adaptations in D1/D2-SPNs in the absence of FMRP (Figure 6).

### Multivariate analysis of the compound effects of *Fmr1* ablation in identified SPNs

**FIGURE 7:**
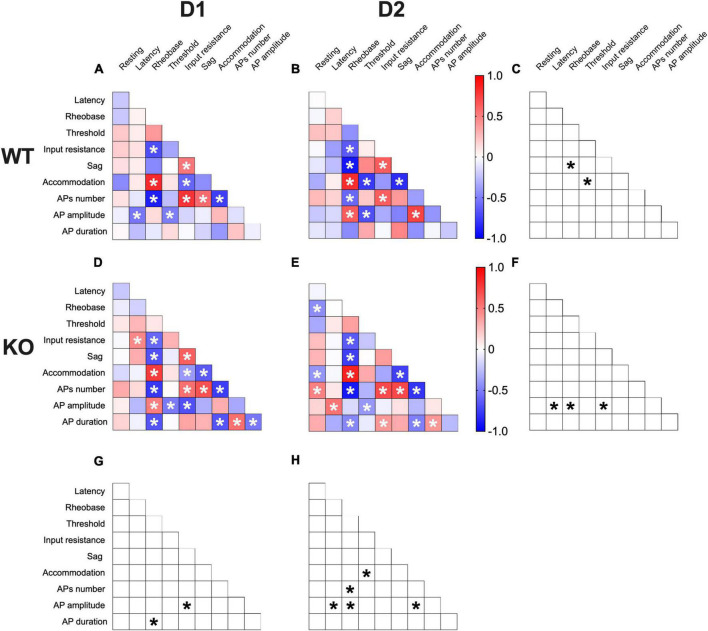
Multivariate analysis of the compound effects of *Fmr1* ablation in identified SPNs’ subtypes. **(A,B)** Heat maps of correlations profiles of electrophysiological parameters of D1- and D2-SPNs in WT or **(D,E)** *Fmr1* KO mice. **(A,B,D,E)** Non-parametric Spearman correlation matrix (*r* values). Statistically significant correlations (*p*-values < 0.05) are displayed in graphs with white *. **(C,F–H)** Correlations that significantly differ (*p*-value < 0.05, Fisher’s *Z* test) are indicated with a black * by **(C,F)** genotype and **(G,H)** cell-type. **(A–H)** WT D1-SPNs *N* = 18, WT D2-SPNs *N* = 16, KO D1-SPNs *N* = 25, and KO D2-SPNs *N* = 25.

Correlations among electrophysiological parameters have important functional implications in neurons. A multivariate analysis was used to evaluate how the lack of FMRP alters the relationships between pairs of electrophysiological features (Figures 7A, B, D, E). In WTs, SPN subtypes notably differed in sag-rheobase and accommodation-threshold correlations (Figure 7C). In contrast, *Fmr1* KO exhibited differences in the correlation between AP amplitude and latency, rheobase, and input resistance (Figure 7F). Across genotypes, D1-SPNs displayed variations in the relationship between AP duration and rheobase, as well as AP amplitude and input resistance (Figure 7G). On the other hand, D2-SPNs showed differences in the correlation between AP amplitude and latency, rheobase, and accommodation, as well as APs number and rheobase, and accommodation and threshold (Figure 7H). These data further illustrate how FMRP deficiency strongly affects SPN subtypes’ electrophysiological profiles.

## Discussion

Fragile X syndrome is the most common monogenic cause of autism and inherited intellectual disability (Protic et al., 2022). Cognitive, emotional, and social deficits observed in FXS patients are compatible with dysfunction in the ventral striatum (Kohls et al., 2013; Gunaydin et al., 2014; Wang et al., 2014; Floresco, 2015; Rogers-Carter et al., 2019; Dai et al., 2022). Although the striatum is an essential component of the autistic phenotype (Fuccillo, 2016), studies have so far focused on the dorsal striatum, leaving the role of its ventral part poorly understood.

The present study provides the first cell-type specific electrophysiological profile of identified accumbal neurons in adult FXS mice. The data show that the functional dichotomy that characterizes WT SPN activity (i.e., D1-SPNs are more excitable than D2-SPNs), disappears in FXS. Thus, FMRP deficiency decreases the excitability of D1-SPNs and increases that of D2-SPNs. Mechanistically, this reversed phenotype is caused by both a more negative threshold and lower input resistance in D1-SPNs and a more depolarized resting potential, lower rheobase, and greater input membrane resistance in D2-SPNs.

Although the canonical separation of D1-direct and D2-indirect pathways in the accumbens is rightly disputed (Kupchik and Kalivas, 2017), the fact remains that D1-SPN and D2-SPN are major contributors to direct and indirect pathways, respectively (Surmeier et al., 2007). Thus, the alteration in excitability between D1 and D2 reported here will alter accumbal outputs: the increased and decreased excitability of D2- and D1-SPNs, respectively, will likely unbalance mesolimbic pathways in favor of the indirect route. In keeping with this hypothesis, clinical and preclinical data both indicated a greater avoidance/approach behavior ratio in ASD, suggestive of an elevated tone of the indirect mesolimbic pathway (Pfaff and Barbas, 2019).

In FXS, channel dysfunctions underlie pathological changes in neuronal excitability. FMRP regulates the gene expression or the function of various channels, thereby modulating AP properties (Deng and Klyachko, 2021). Investigated AP kinetics in FXS mice showed that the absence of FMRP increased and decreased the amplitude of APs in D1- and D2-SPNs, respectively. Additionally, AP duration was shorter in D1- and longer in D2-SPNs of FXS mice than in WT littermates. These changes in AP kinetics result from mirroring alterations in depolarization and repolarization phases, giving further credit to the idea that *Fmr1* ablation is linked to cell-specific modifications in the NAc. In D1-SPNs of FXS mice, repolarization was normal, but depolarization was shorter, in agreement with the known effects of FMRP ablation on voltage-gated Na^+^ channels (Deng and Klyachko, 2021). D2-SPNs displayed longer depolarization and repolarization than WT. These changes are reminiscent to those reported in cortical and hippocampal neurons where the lack of FMRP influences big potassium (BK) channel conductance causing AP broadening and a higher firing probability (Deng et al., 2013). Thus, a difference in BK activity could explain the altered repolarization observed in D2-SPNs.

Multivariate analysis uncovered covariation of selected intrinsic properties in a cell-specific manner in the absence of FMRP and suggested a degree of functional dependence in the associated neuronal properties. While some parameters covariate, keeping their correlation in all groups (e.g., rheobase-AP number), other parameters correlate differently across cell-types and genotypes (e.g., AP amplitude with latency, rheobase, input resistance, and accommodation). Dopaminergic control of accumbal D1- and D2-SPNs is central to reward behaviors (Bromberg-Martin et al., 2010). DA D1R-mediated enhancement of L-type Ca^++^ channels augments D1-SPNs excitability while binding of D2R has the opposite action in D2-SPNs (Surmeier et al., 1995; Hernández-López et al., 1997; Hernádez-López et al., 2000). FMRP is a key messenger for dopamine modulation in the striatum (Wang et al., 2008) and consequently dopamine signaling is altered in FXS mice (Kosillo and Bateup, 2021). It is tempting to hypothesize that the cell-type specific perturbation of excitability in D1- and D2-SPNs reported here results, at least partly, from dopaminergic dysfunctions in the NAc of FXS mice.

### Limitations

In our opinion, there are three main limitations to the present study. First, although females with FXS show a high frequency of mood and learning disorders, and are more vulnerable to social anxiety and avoidance, the neuronal phenotypes of NAc neurons in female were not considered in this study, due to time and funding limitations. Thus, the study could benefit from considering female mice. This would clarify the contribution of sex to the phenotypic differences reported here. Second, SPN’s activity was purposely studied here in the presence of a GABA-A antagonist, to isolate SPNs from inhibitory GABA synaptic inputs. Nonetheless, *in vivo* NAc neurons are innervated by strong GABA inputs from various subtypes of interneurons and SPNs themselves. The ensuing inhibitory bombardment could differentially affect the firing patterns described in the current study and recordings in the absence of would allow us to assess the relative contribution of tonic inhibition on SPN’s activity. Third, although synaptic inhibition and excitation are basic functional principles in the CNS, we limited our *ex vivo* functional exploration to intrinsic properties of identified SPN. Optogenetically disambiguated recording of inhibitory GABA and/or glutamatergic synaptic inputs would provide insight into how FXS alters synaptic networks.

## Conclusion

In conclusion, these results show that the absence of FMRP induces a cell-specific phenotype of SPNs in the NAc and further emphasize the need to study the role of NAc in this disease.

## Data availability statement

The raw data supporting the conclusions of this article will be made available by the authors, without undue reservation.

## Ethics statement

Animals were treated in compliance with the European Communities Council Directive (86/609/EEC) and the United States National Institutes of Health Guide for the care and use of laboratory animals. The French Ethical Committee authorized this project (APAFIS#3279-2015121715284829 v5).

## Author contributions

GG: conceptualization, data curation, formal analysis, validation, and writing—review and editing. OL: data curation, formal analysis, validation, and methodology. LM-M: data curation, formal analysis, and validation. EV: data curation, formal analysis, validation, and writing—review and editing. PC: conceptualization, supervision, and methodology. OM: conceptualization, supervision, funding acquisition, methodology, project administration, and writing—original draft, review, and editing. All authors contributed to the article and approved the submitted version.
