# Supplementary material for: Investigating cell-specific effects of FMRP deficiency on spiny projection neurons in a mouse model of Fragile X syndrome
Source: Front Cell Neurosci. 2023 May 30;17:1146647. doi: 10.3389/fncel.2023.1146647 (PMC10264852; doi:10.3389/fncel.2023.1146647)
Supplement: Supplementary file 1 [file Data_Sheet_1.docx]

**
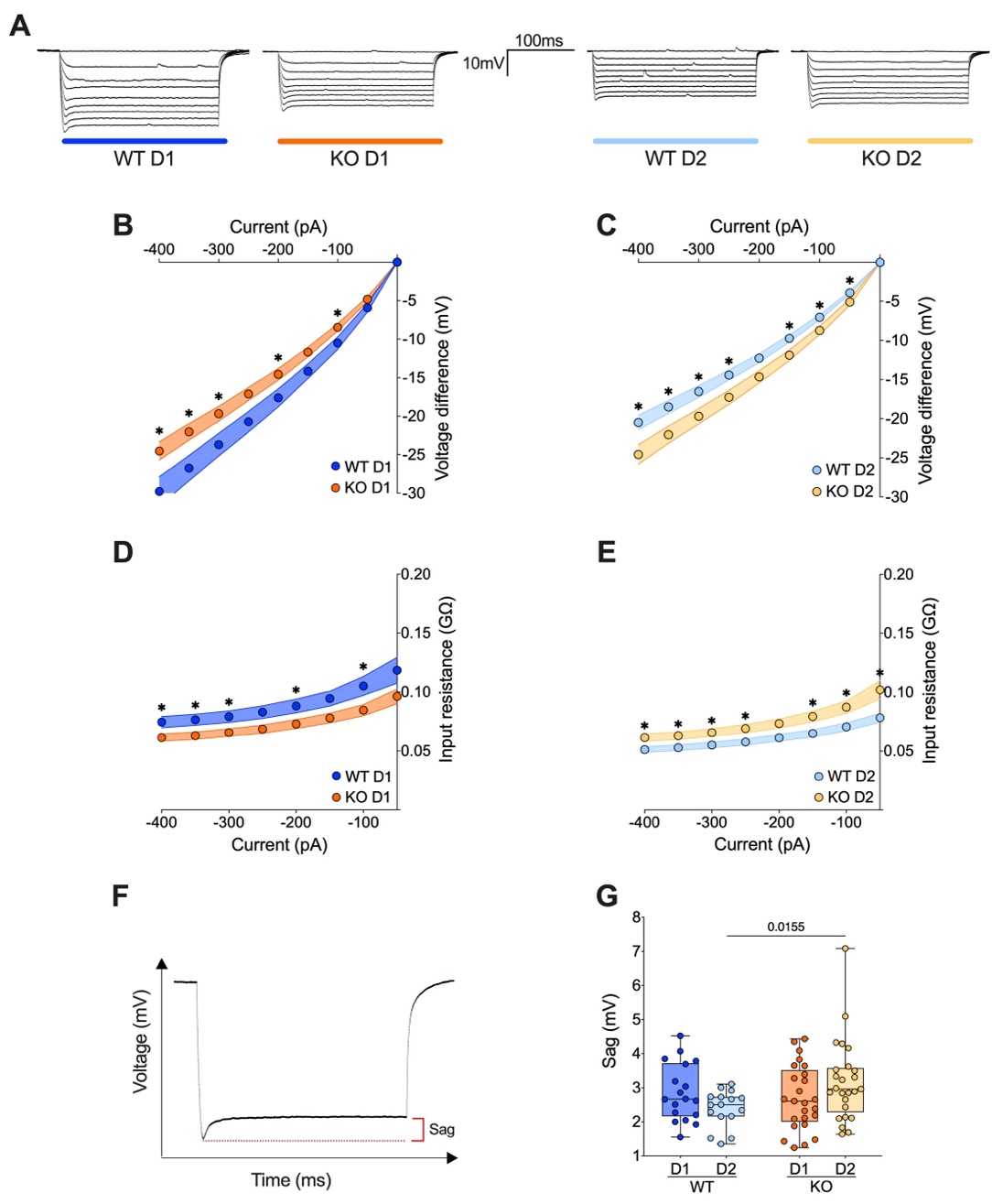
**

**Supplementary Figure 1.** **Lack of FMRP induces cell-specific alterations in the membrane voltage response to hyperpolarizing current injections.**

**(A)** Examples of membrane voltage response to hyperpolarizing current injections in our experimental groups. **(B)** In response to hyperpolarizing current injections D1-SPNs membrane voltage response is greater in WT than *Fmr1* KO mice. **(C)** On contrary, D2-SPNs show a lower response in WT than *Fmr1* KO mice. **(D)** Accordingly, in absence of FMRP, D1-SPNs have a higher input resistance, **(E)** while D2-SPNs have a lower one. **(B-E)** Single dot represents group mean value at that current step. Data are shown as mean ± SEM in XY plot. Multiple Mann-Whitney *U* test. * p-values <0.05. **(F)** Example of membrane voltage response to a -400pA current injection, indicating the sag voltage. **(G)** In response to a -400pA current injection D2-SPNs exhibit a smaller sag voltage in WT compare with FXS mice. Single dot represents an individual mouse. Data are shown as min. to max. box plot with median and 25-75 percentile. Mann-Whitney *U* tests. P-values <0.05 are displayed in graphs. **(B-E, G)** WT D1-SPNs N=18 in dark blue, WT D2-SPNs N=16 in light blue, KO D1-SPNs N=25 in dark orange, KO D2-SPNs N=25 in light orange.

**
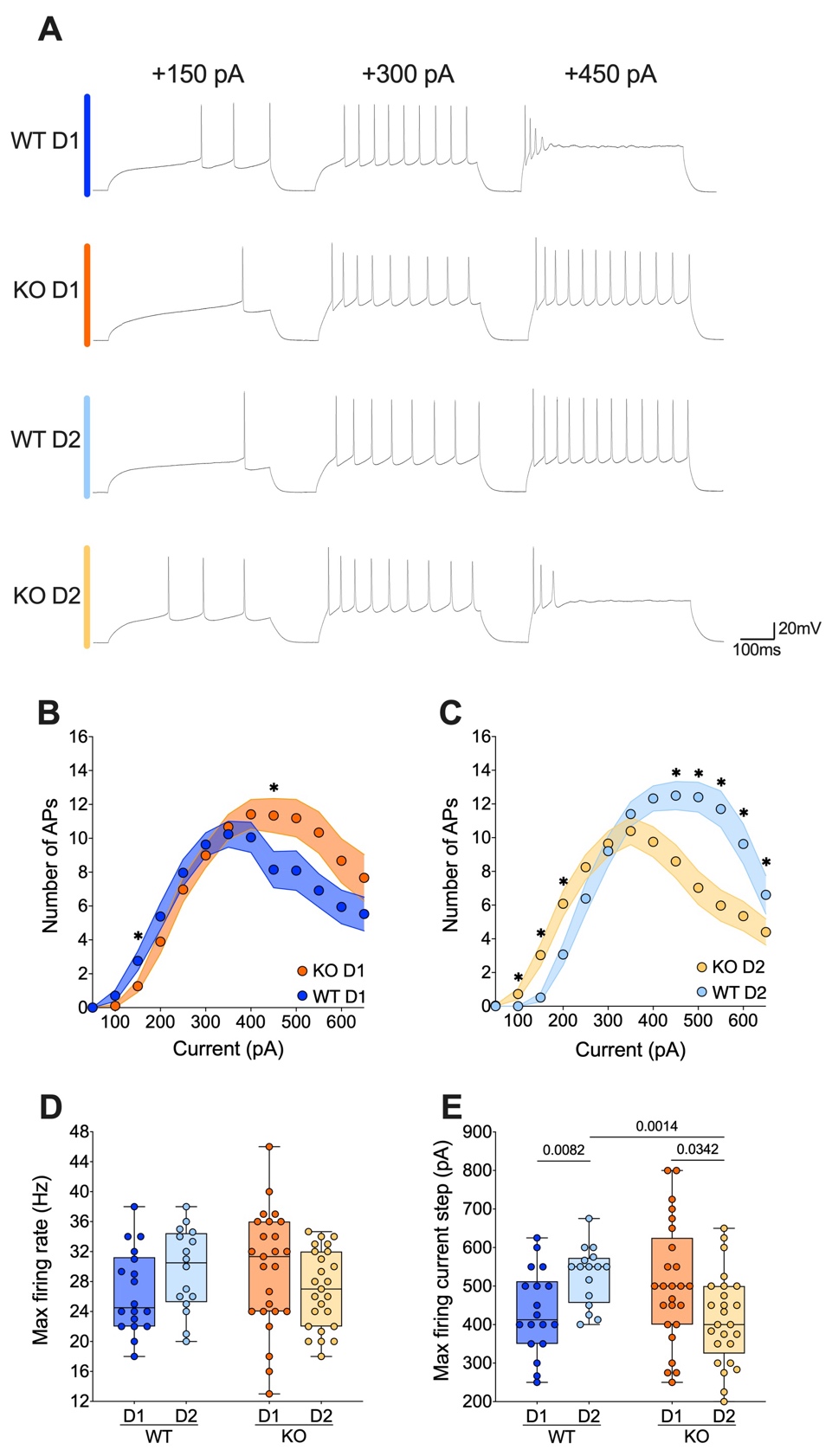
**

**Supplementary Figure 2. In absence of FMRP D1- and D2-SPNs excitability change in opposite.**

**(A)** Example of firing pattern triggered by the injection of +150pA, +300pA and +450pA depolarizing currents for each group. **(B, C)** Analysis by cell-type of the number of evoked action potentials in response to increasing depolarizing current shows that FMRP absence has an opposite effect on D1- and D2-SPNs firing profile. Single dot represents group mean value at that current step. Data are shown as mean ± SEM in XY plot. Multiple Mann-Whitney *U* test. * p-values <0.05. **(D)** No statistically significant difference was found in the maximum firing rate achieved by SPNs during increasing steps of depolarizing currents. **(E)** Analysis of the amount of current required to induce the higher firing rate showed that in WT mice D2-SPNs reached their maximum firing at higher current steps than D1-SPNs, whereas it was the opposite between SPNs of FXS mice. **(D, E)** Single dot represents an individual mouse. Data are shown as min. to max. box plot with median and 25-75 percentile. Mann-Whitney *U* tests. P-values <0.05 are displayed in graphs. **(B-E)** WT D1-SPNs N=18 in dark blue, WT D2-SPNs N=16 in light blue, KO D1-SPNs N=25 in dark orange, KO D2-SPNs N=25 in light orange.
